# Supplementary material for: Universal Guide for Skull Extraction and Custom-Fitting of Implants to Continuous and Discontinuous Skulls
Source: eNeuro. 2022 Jun 17;9(3):ENEURO.0028-22.2022. doi: 10.1523/ENEURO.0028-22.2022 (PMC9215690; doi:10.1523/ENEURO.0028-22.2022)
Supplement: Extended Data — Example CAD models and implants. Download Extended Data, DOCX file. [file enu-eN-MNT-0028-22-s02.docx]

**Extended Data Table 1-1**

| Software name | Version number | Links |
| --- | --- | --- |
| 3D slicer | 4.8.1 (2019) | ‘FastGrowCut’  <https://github.com/Slicer/Slicer/tree/v4.8.1> |
| 3D slicer | 4.10.1 (2019) | ‘GrowFromSeeds’  <https://github.com/Slicer/Slicer/tree/v4.10.1> |
| Fusion 360 | Downloaded 2019 | <https://www.autodesk.de/products/fusion360/overview> |
| Rhinoceros 5 | Downloaded on Windows (2018) | <https://www.rhino3d.com/de/download/rhino/5/latest/> |
| Rhinoceros 6 | Downloaded on Mac (2019) | <https://www.rhino3d.com/download> |
| Meshmixer | 3.5 | <https://www.meshmixer.com/> |
| MeshLab | 2016 | <https://www.meshlab.net/> |
